# Congress report of the 23^rd^ AGE annual meeting from 26^th^ - 28^th^ April 2018 in Hamburg

**DOI:** 10.4274/jtgga.2018.0076

**Published:** 2018-08-06

**Authors:** İbrahim Alkatout, Bernd Holthaus

**Affiliations:** 1 Department of Obstetrics and Gynecology, University Hospitals Schleswig-Holstein, Campus Kiel, Kiel, Germany; 2 Clinic of Obstetrics and Gynecology, St. Elisabeth Hospital, Damme, Germany

**Keywords:** Congress, report

## Abstract

The Study Group of Gynecological Endoscopy (AGE) has a growing number of members each year. This is an acknowledgment as well as a challenge for the study group. The challenges were faced in the form of exemplary cooperative work by the core members of AGE, the Velen Study Group for Ambulant Surgery (VAAO), the Foundation of Endometriosis Research (SEF), the Study Group of Urogynecology and Plastic Pelvic Floor Reconstruction (AGUB), the Study Group for Robotic-assisted Surgery in Gynecology (ARC^Gyn^), and the Study Group of Gynecological Oncology (AGO). More than 1500 AGE members have been able to create significant effects preemptively by designing a Congress program that was prepared interactively. The program of live surgery was designed in the course of two days on the basis of an online inquiry. The first transmission of laparoscopy on a body donor and anatomic demonstrations on formalin-fixed specimens were especially significant in this context. Sessions of general gynecology, including myoma therapy, endometriosis and infertility treatment, and gynecologic oncology and urogynecology covered the entire spectrum of minimally invasive surgical techniques. Individual topics were addressed in specific courses. The Congress was preceded by an optional certified basic course (MIC I) of the AGE. Far more than 500 congress attendees from all German-speaking countries were spirited away to a paramedical steep face, which was ascended together with a renowned German extreme climber. The keynote lecture was especially impressive and held by the pioneer and founder of the neuropelveology. The world’s leading expert in this field described the responsibilities of our specialty in a visionary manner and motivated all of the listeners strongly in regard of their actions and efforts.

## Program

The Study Group of Gynecological Endoscopy (AGE) registers a growing number of members each year. This is an acknowledgement as well as a challenge. It is an acknowledgement of the fact that the needs of its members have been fulfilled satisfactorily over the years. At the same time, the growing technical and formal demands of the German and international gynecologic societies in terms of clinical, scientific and training activities could also be fulfilled. However, it is also a challenge because rapid and complex developments in medicine, medical technology, and the pharmaceutical industry present major responsibilities and tasks in clinical routine. Furthermore, the increasing level of enlightenment among patients and their sense of entitlement, as well as the low level of tolerance in terms of medicolegal action, have become the focus of our actions.

**Figure 1:**
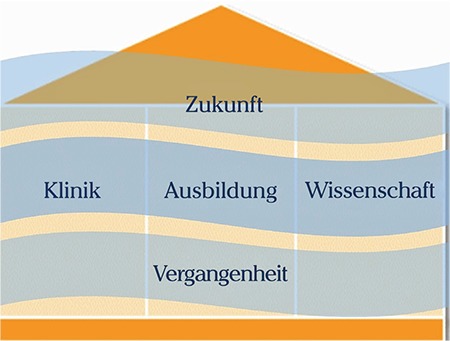
Concept of the three essential pillars - health services at the clinic, training, and science - which have always onstituted the foundations of our medical work and will always do so in the future. Training is one of the essential concerns of the Study Group of Gynecological Endoscopy, indivisibly linked with health care and science, anchored in the experiences of the past and the visions of the future

The 23^rd^ Annual Meeting of the Study Group of Gynecological Endoscopy (AGE) was held from April 26^th^ to 28^th^, 2018, at the Radisson Blue Hotel in Hamburg. The Congress marked the 25^th^ anniversary of the founding of the study group. Some aspects of the program were based on the three essential pillars of a doctor’s life, analogous to the integration of conservative and innovative methods in hybrid technology (Figure 1).

**Figure 2:**
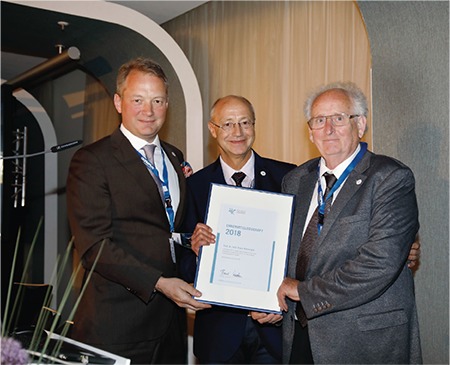
The president of the German Society for Gynecological Endoscopy (left) hands over the honorary membership to two laureates

After the inauguration of the Congress by Dr. Bernd Holthaus, Congress President and First Chairman of the AGE, and Prof. Diethelm Wallwiener, Past President of the AGE and the German Society of Obstetrics and Gynecology (DGGG), two people were awarded an honorary membership. The first of these was Prof. Klaus Kolmorgen who was commended for his lifework and his commitment to minimally invasive surgery (1,2). In his laudatory speech, Dr. Rüdiger Müller from Königswusterhausen emphasized Dr. Kolmorgen’s pioneering work in the era of the German Democratic Republic (Figure 2).

The second honorary membership was awarded post mortem to the Past President of the AGE, Associate Professor Thoralf Schollmeyer. In an evocative and emotional speech, Dr. Liselotte Mettler reminded us of the significance of Thoralf Schollmeyer’s achievements for the AGE and the continuation of the Semm legacy at the Kiel School of Gynecological Endoscopy in Kiel (3,4,5).

## Golden Scope Science Prizes

The Golden Scope (sponsored by Karl Storz Company) for outstanding achievements in gynecologic endoscopy was awarded to Dr. Claus Peter Möller from Hamburg. Claus Möller was Acting President of the AGE from 2012 to 2013.

The science prizes are listed in Table 1.

## Key Science Aspects of the AGE Annual Meeting

### 1. MIC – Basic Course

At the preliminary program of the actual Annual Meeting, an MIC Basic Course certified by the AGE was held under the leadership of the AGE training centers of Damme, Hamburg and Kiel. On great demand, 30 attendees were supervised most competently by staff members of the three training centers as well as Karl Storz and Richard Wolf Company. The attendees had the opportunity to practice on the traditional pelvitrainer as well as the virtual hysteroscopy trainer. The basic course was headed by Peter Biel in a familiar atmosphere.

### 2. Intensive Courses

Without complication upon the schedule of the main program, 8 courses, which focused on individual topics, were held for small groups. Selected tutors made it possible for colleagues to exchange their knowledge and gain close insights into specialized fields.

      -         Reproduction Medicine and Surgery

      -         Multidisciplinary Surgery

      -         VAAO and Hysteroscopy

      -         Complication Management

      -         Complex Operations Explained Step by Step

      -         Myoma Treatment

### 3. Scientific Sessions

In line with the trend towards highly individualized yet strictly guideline-based treatment concepts, the main program offered scientific sessions on the subjects of general gynecology, (including myoma, endometriosis and infertility treatment), gynecologic oncology, and urogynecology.

The session on General Gynecology addressed the procedure for the treatment of complex myomas, a niche after caesarean sections and hysteroscopy techniques, and the session on Urogynecology was focused on current problems in descensus and incontinence. The session was held jointly by the AGE and the Study Group of Urogynecology and Plastic Pelvic Floor Reconstruction (AGUB). Core aspects included the differentiation between vaginal and laparoscopic access, the treatment of recurrent disease after mesh placement, and the avoidance of complications following extensive ultrasound diagnostic investigation. The session on endometriosis, held jointly with the Foundation of Endometriosis Research, presented current efforts to impose meaningful limitations on extensive surgery (6).

The session on Laparoscopy in Oncology was held together with the Study Group of Gynecological Oncology (AGO). Here the role of laparoscopy in oncology was addressed in detail, along with the current body of data on the subject. Options for the use of laparoscopy in ovarian cancer, its role in endometrial cancer, the role of the robot, and the technique of compartment-based surgery founded by Höckel (7,8) were discussed. It became evident that endoscopy plays a role in all relevant malignant diseases. It is the gold standard for endometrial cancer, and its role in cervical cancer is being discussed anew because of recent data concerning the prognosis of the disease in patients who undergo endoscopic versus open surgery. In ovarian cancer, endoscopic surgery is regarded internationally as an option for staging, along with the use of preoperative reaction scores (9).

The session on hysterectomy (10) was focused on the extent of resection, access in borderline situations, and the question of in-bag morcellation systems (11,12). The session ‘My Special Case’ was very well accepted despite the advanced time of the day. The purpose of the session was to conduct an open discussion of complications and their management, as well as exceptional cases and their specific challenges.

The scientific sessions were complemented by industrial symposia on the subjects of fluorescence-based diagnostic imaging (indocyanine green) by Olympus Company, myoma therapy (Gedeon Richter), and hysteroscopy (Karl Storz).

### 4. Scientific highlights of the last day of the Congress

The last day of the Congress was focused on training but was as well attended as the preceding days, thus reflecting the attendees’ sense of responsibility towards future generations. Videos of operations were presented at this session. Entire operation sequences were re-edited and accompanied by live commentaries. The didactic framework offered adequate opportunity for the exchange of knowledge. The session included a panel discussion on training, which was marked by a lively discussion on the concerns of advanced training assistants.

### 5. Live surgery on two days at two locations

**Figure 3:**
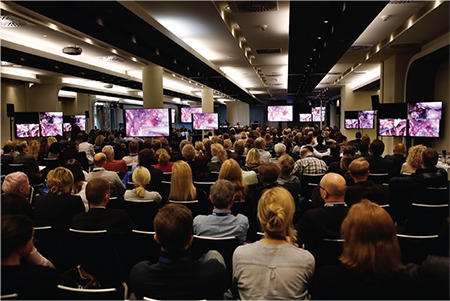
Impressions during the live transmission

Live transmissions are the soul of the annual meeting of a society focused on surgery. Therefore, the time for live transmissions was extended to two days by the Congress Committee, but there were fewer operations than in the past. For the first time, all AGE members participated actively in planning live operations. Prior to the Congress, all AGE members were sent an online questionnaire and requested to join in the selection of the operations. More than 30% responded. Thus the attendees could indirectly influence the surgery program. The result was a surgery program consisting of urogynecologic, oncologic, and laparoscopic operations for myoma and endometriosis as well as hysteroscopic operations. On April 26^th^ and 27^th^, robotic-assisted operations were transmitted for the first time at an annual meeting of the AGE, in addition to laparoscopic surgery (Figure 3) (13).

**Figure 4:**
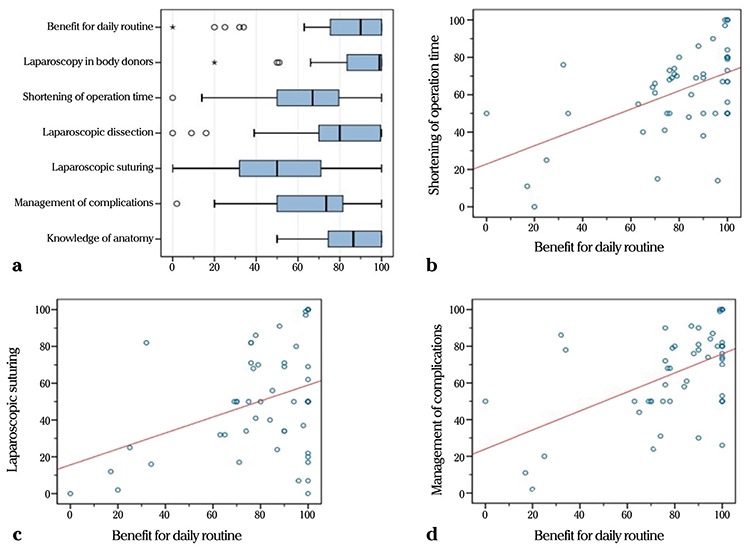
Results of a face validation survey among members of the training course on Laparoscopy. The results (a-d) confirm that a laparoscopic training course helps the attendees in all essential aspects of their daily work (such as operating time, suture techniques, and complication management)

At the live transmissions, experienced surgeons demonstrated the innovative management of various diseases, taking the most recent developments in medical technology into account. This included, in an educational atmosphere, an exchange of information with the attendees in the auditorium. Based on the medical-ethical principles of autonomy, beneficence, non-maleficence, and justice (righteousness, fairness, justness), this approach was reviewed critically and repeatedly; possible equivalent alternatives to this approach were discussed (Figure 4) (14).

All the same it should be noted that the benefits of training and teaching under safe medical and ethical conditions have been comprehensively established in clinical and anatomic curricula (15,16,17,18).

**Figure 5:**
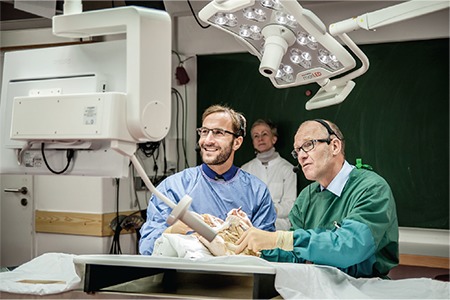
Live demonstration of formalin-fixed body donors into the auditorium of the AGE Annual Meeting in Hamburg. This figure shows the topographical anatomy in the lesser pelvis *AGE: Gynecological Endoscopy*

**Figure 6:**
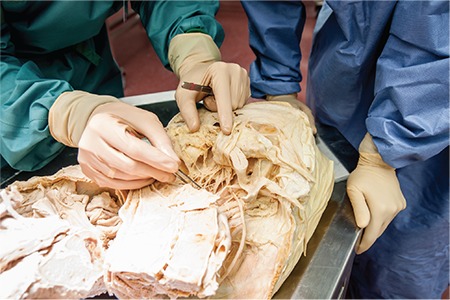
Live demonstration of formalin-fixed body donors into the auditorium of the AGE Annual Meeting in Hamburg. This figure shows the course of vessels in the lesser pelvis *AGE: Gynecological Endoscopy*

For the first time at a live surgery demonstration in Germany, a body donor was investigated by laparoscopy. For the purpose of teaching, this anatomic presentation was complemented by a video demonstration of selective formalin-fixed specimens (Figure 5, 6).

The implementation and transmission of the operations were rendered possible by the assistance of Karl Storz SE & Co. KG Company, Olympus Deutschland Private Limited Company, Richard Wolf, and Intuitive Surgery, and were executed technically by TV-Studios Leonberg Company.

## Major non-scientific aspects of the AGE Annual Meeting

### 1. Keynote Lecture Alexander Huber

**Figure 7:**
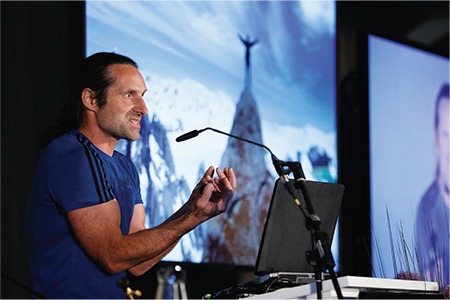
The German extreme climber in his element - the unification of man and mountain

Analogy of mountaineering and work. The professional mountaineer and extreme climber Alexander Huber (www.huberbuam.de) addressed subjects like motivation, courage, creativity, planning, risk and risk management in a uniquely comprehensible manner with the aid of impressive pictures, videos and personal experiences (Figure 7).

These subjects constitute everyday challenges or play a key role in the medical profession as well, but medical professionals are rarely prepared for these challenges in an adequate manner.

### 2. Keynote lecture of Mark Possover

**Figure 8:**
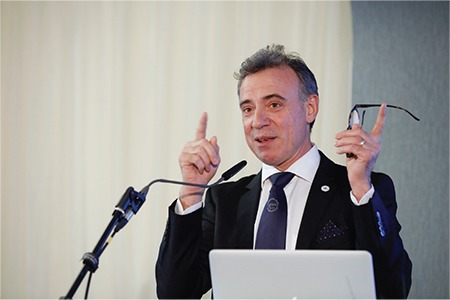
The pioneer and founder of the neuropelveology appeals to the attendees’ individual sense of responsibility and the practical application of the Hippocratic Oath in our times; he raises both hands upward while doing so

A very special highlight was Mark Possover’s keynote lecture, which provided an overview, a retrospective view, and a future view of the options of neuropelveology. His creative work is rooted in the AGE and in gynecological endoscopy. Through his extraordinary vision, pioneering spirit and courage, Professor Mark Possover was able to expand his therapies to include the treatment of the loss of spinal cord functions. As a visionary, he eventually led the entire auditorium into spheres that were known so far only in the field of aerospace technology (Figure 8) (19,20).

The next Congress will be held in 2020 in Berlin under the leadership of the designated President Prof. Dr. Uwe A. Ulrich.

## Figures and Tables

**Table 1:**
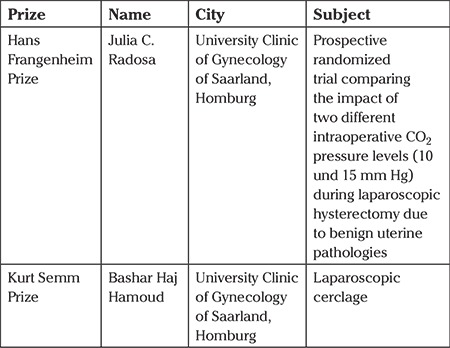
Science Prizes at the 25^th^ Annual Meeting of the AGE from 26 to 28 April 2018 in Hamburg
